# Long-term ground deformation patterns of Bucharest using multi-temporal InSAR and multivariate dynamic analyses: a possible transpressional system?

**DOI:** 10.1038/srep43762

**Published:** 2017-03-02

**Authors:** Iuliana Armaş, Diana A. Mendes, Răzvan-Gabriel Popa, Mihaela Gheorghe, Diana Popovici

**Affiliations:** 1 University of Bucharest, Faculty of Geography, Department of Geomorphology-Pedology-Geomatics, Nicolae Balcescu 1, Sector 1, 010041, Bucharest, Romania; 2 ISCTE-IUL and BRU-IUL, Department of Quantitative Methods for Management and Economics, Avenida Das Forças Armadas, 1600-083, Lisbon, Portugal; 3 Institute of Geochemistry and Petrology, ETH Zürich, Clausiusstrasse 25 NO, 8092 Zürich, Switzerland; 4 Technical University of Civil Engineering of Bucharest, Faculty of Geodesy, 124 Lacul Tei Boulevard, 020396 Bucharest, Romania

## Abstract

The aim of this exploratory research is to capture spatial evolution patterns in the Bucharest metropolitan area using sets of single polarised synthetic aperture radar (SAR) satellite data and multi-temporal radar interferometry. Three sets of SAR data acquired during the years 1992–2010 from ERS-1/-2 and ENVISAT, and 2011–2014 from TerraSAR-X satellites were used in conjunction with the Small Baseline Subset (SBAS) and persistent scatterers (PS) high-resolution multi-temporal interferometry (InSAR) techniques to provide maps of line-of-sight displacements. The satellite-based remote sensing results were combined with results derived from classical methodologies (i.e., diachronic cartography) and field research to study possible trends in developments over former clay pits, landfill excavation sites, and industrial parks. The ground displacement trend patterns were analysed using several linear and nonlinear models, and techniques. Trends based on the estimated ground displacement are characterised by long-term memory, indicated by low noise Hurst exponents, which in the long-term form interesting attractors. We hypothesize these attractors to be tectonic stress fields generated by transpressional movements.

At present, when global environments are increasingly becoming urbanised, sustainable growth and development should be based on a good understanding of the long-term ground displacement dynamics. The issue of ground displacements in urban areas is of great importance for the development of cities, due to possible instabilities induced by subsidence and uplift. When human interaction perturbs displacement trends with natural causes that act on a long time-scale, the issue gains even more weight^1^,^2^,^3^. In earthquake-prone environments, understanding natural displacement patterns is even more important because they could become latent risk factors. Since Bucharest, the most earthquake-prone capital in the European Union, is often compared to Mexico City in terms of seismic hazards caused by distant earthquakes that affect tall buildings, understanding long-term ground displacement trends is an important task^4^,^5^. Identifying ground deformation trends in urban areas is also a continuous challenge for researchers when lacking technological support of high-precision, repeated, and large-scale ground displacement measurements.

After 2001, identifying ground displacement dynamics in urban areas has been supported by high-precision, repeated, and large-scale measurements of ground displacements using InSAR techniques^6^,^7^,^8^,^9^,^10^. Some of these studies focused on subsidence, which sometimes measured less than 1 mm and was caused by groundwater over-exploitation, or uplift^11^,^12^,^13^,^14^,^15^. Regarding Bucharest, conventional and radar interferometric techniques have been recently used to detect and monitor ground displacement^16^,^17^,^18^,^19^,^20^,^21^ and Digital Elevation Model (DEM) extraction^22^,^23^.

This research aims to identify trends in Bucharest’s ground displacement dynamics by analysing three sets of synthetic aperture radar (SAR) satellite data collected over a period of 20 years: historical European Space Agency (ESA) SAR data acquired by European Remote Sensing (ERS-1/-2) and ENVISAT satellites and recent high-resolution German Aerospace Center (DLR) SAR data acquired by the TerraSAR-X (TSX) satellite (Fig. 1). InSAR estimates were compared to diachronic analyses based on detailed historical maps (i.e., Borroczyn map, 1852; Szatmary map, 1864; the Bucharest City Plan, 1911, 1921, 1940; topographical map, 1980) and orthophotomaps (2006, 2008, and 2010), ground measurements, and traditional geological and geomorphological investigations. The results suggest consistent displacement patterns over the city.

## Ground Movement Assessment

### SAR Satellite Data

Historical satellite data consisted of 28 ERS-1/-2 images acquired between 1992 and 1999 and 24 ENVISAT images captured between 2003 and 2010 (© ESA, 2012). Both ERS-like and ENVISAT data were acquired on Track: 465 and Frame: 2709. Additionally, 27 high-resolution single-polarisation X-band images were collected by the TSX satellite between 2011 and 2014.

These images were carefully selected from a larger pool of procured SAR images. SAR images were selected for multi-temporal interferometric synthetic aperture radar (InSAR) analysis by eliminating datasets acquired during snowfall or with snow groundcover, during rain, thunderstorms, and overcast conditions. The weather information was extracted from historical local observations, made available with a half an hour frequency on www.wunderground.com. Datasets with large perpendicular baselines, especially when associated with one of the atmospheric conditions listed previously, were also eliminated.

### Multi-Temporal InSAR Analysis

High-resolution multi-temporal InSAR techniques, specifically Persistent Scatterers (PS) InSAR^24^,^25^ and the Small Baseline Subset (SBAS) InSAR^26^ have been applied to both historical (i.e., ESA) and recent (i.e., DLR-German Aerospace Centre) SAR satellite data. The steps used in the PS InSAR and SBAS InSAR analyses followed specifics described in Armaş *et al*.^21^, and Necşoiu and Walter^27^. The choice of using both techniques relies on the fact that the PS InSAR applicability is limited to temporally uniform rates of displacement, while SBAS InSAR has the ability to capture strong nonlinearities in the study area. Unlike PS InSAR, the SBAS InSAR technique does not coherently ‘see’ point targets, but generates highly correlated areas in time that help derive the deformation signal from multi-looked interferograms. In the SBAS InSAR concept, data pairs involved in the generation of interferograms are properly selected to minimize the spatial and temporal separation between the acquisition orbits, thus mitigating the spatial and temporal decorrelation^28^. All SAR raw datasets were imported in SARscape^®^ software 5.1 from SARMAP^®^ and focused (i.e., radar-dispersed energy was focused into single pixels using data compression in both range and azimuth directions)^29^ to generate Single Look Complex (SLC) datasets. For each stack of SAR datasets (i.e., ERS, ENVISAT, TSX), a master image was selected based on minimizing the perpendicular and temporal baseline of the satellite. Interferograms are then formed by phase differencing between two acquisitions. For PS InSAR, the interferograms result from one master image and the rest of the slave images. In the case of the SBAS technique, more than one master image is coupled with slave images based on the spatial and temporal baselines between each of them. In both cases, a digital elevation model (DEM) was used to remove the initial topographic phase. Our DEM was based on six one-arc-second X-band DEM products^30^ covering Bucharest and the surrounding areas, and was constructed and used in analyses. The next step was obtaining the interferogram coherence through filtering in the case of SBAS, or the coherent radar signal reflectors for PS. For each coherent radar targets, the phase history is analysed and good candidates are extracted for deriving the residual height and displacement history through a first inversion. A second inversion helps removing the components of the atmospheric influence from the products obtained in the previous step and derives displacement time series. Finally, the following products were generated:

■ Mean displacement velocity

■ Displacement history, which consists of the displacement magnitude for each input file with respect to the reference acquisition

■ Measurement precision, for both mean displacement velocity and height

■ Height correction with respect to the input DEM

■ Total displacement

In the case of the displacement maps produced using TSX data, validation based on traditional geological and geomorphological investigations, as well as comparison with field Global Navigation Satellite Systems (GNSS) data was possible. The validation was done by projecting the GNSS three-dimensional coordinates obtained in 14 survey sessions on the LOS direction. The coordinates of each of the 18 GNSS points and 2 permanent stations projected on the LOS direction were compared to the displacement values shown by points located within 100 m^31^. The ERS and ENVISAT data were validated through comparison with movement trends for control areas resulted from TSX. In addition, the results were validated with diachronic cartography, geological and geomorphological research which confirmed that the movement trends described by the SAR historical data correspond to natural or human inflicted displacement trends in the tested areas.

## Displacement Patterns Detection

### Identifying and Selecting Specific Areas of Interest

The maps obtained by multi-temporal InSAR techniques were capable of revealing very small deformation rates (e.g., 1 mm/year), allowing a synoptic view of land deformations in Bucharest. The displacements results obtained by the different sensors are relative to a reference area. In order to compare the obtained results to each other, we have selected the same reference area for each dataset. The area was considered stable from a geological and geomorphological point of view and the PS InSAR processing of the ERS data returned a density of approximately 1500 PS/km^2^ between 1992 and 2000. The processing of the ENVISAT data returned a density of 3000 PS/km^2^ for the period between 2003 and 2010. The majority of points are located in the center of the urban area, but there are also points that cover the periphery and the peri-urban areas. The processing of TSX data identified a higher density of Persistent Scatterers (i.e., 30000 PS/km^2^) when compared to ERS and ENVISAT data processing.

**Figure 2:**
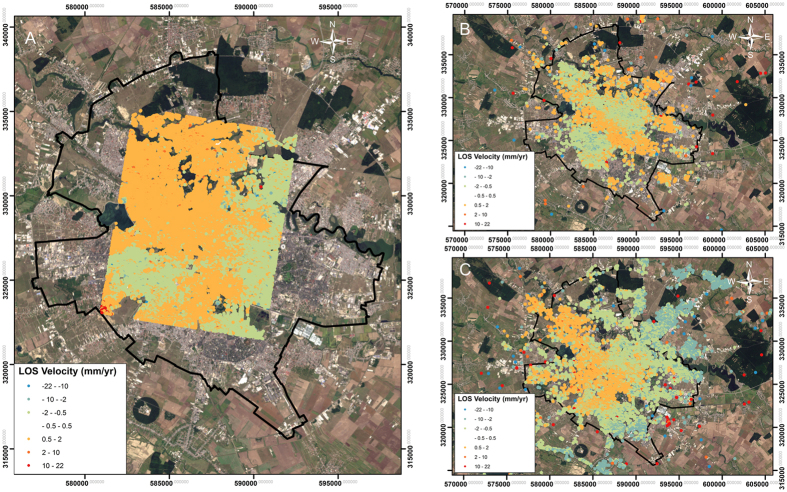
PS InSAR-estimated ground deformation rates in Bucharest from 2011–2014 for TerraSAR-X satellite data (**A**) and 1992–1999 ERS1/2 and 2003–2010 ENVISAT satellite data (**B** and **C**; Data provided by the European Space Agency, 2012). Subsidence is represented by negative displacement values, showing movement away from the satellite. The administrative limit of the city is marked in black. PS and points were processed in SARscape^®^ software 5.1 from SARMAP^®^. Final maps were created in Esri^®^ArcMap™ 10.2. Base map: Landsat-8 scene from 26/08/2015, path 183, row 183, downloaded for free from Earth Resources Observation and Science (EROS) Center, USGS (http://earthexplorer.usgs.gov/; accessed 02/01/2016).

The displacement maps based on historical data revealed stable conditions over most of the city during each time interval, with the majority of points having velocities between −2 and 3 mm/year. Most of the PS showed velocity values between −3 and −1.5 mm/year for 1992–1999, −1.5 and 2.5 mm/year for 2003–2010, and −2 and 3 mm/year for 2011–2014 (Fig. 2). The estimated PS velocity precisions resulted from an uncertainty analysis on our obtained products (~0.3 mm/yr for ERS and ENVISAT and ~0.2 mm/yr for TSX InSAR products).

**Figure 3:**
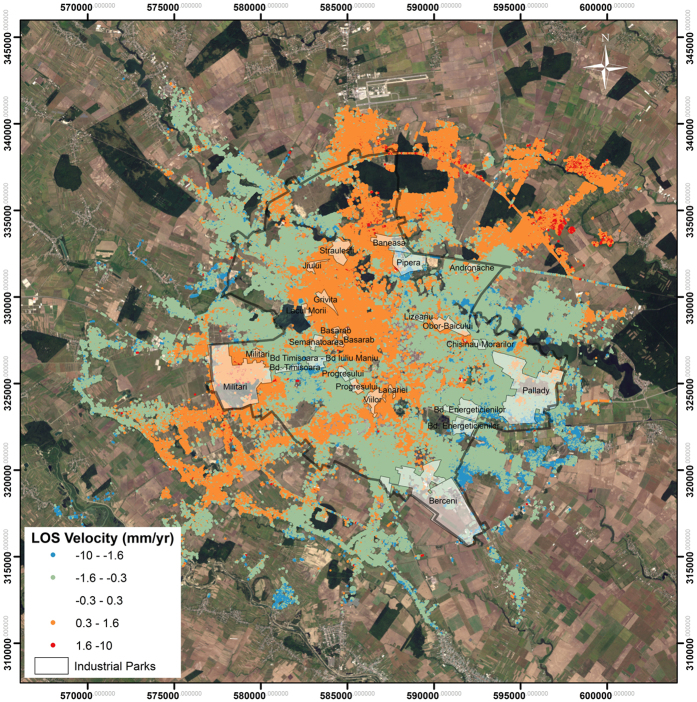
SBAS InSAR ground deformation rates in Bucharest in 2011–2014 for TerraSAR-X satellites (Data provided by the German Space Agency, 2014). The administrative limit of the city is marked in black. Subsidence is represented by negative displacement values, showing movement away from the satellite. PS and points were processed in SARscape^®^ software 5.1 from SARMAP^®^. Final map was created in Esri^®^ArcMap™ 10.2. Base map: Landsat-8 scene from 26/08/2015, path 183, row 183, downloaded for free from Earth Resources Observation and Science (EROS) Center, USGS (http://earthexplorer.usgs.gov/; accessed 02/01/2016).

The SBAS InSAR technique applied to the TSX data returned the same deformation pattern as the PS InSAR analysis, with a difference in displacement values, which are smaller than those depicted from PS InSAR. The SBAS InSAR results featured displacement rates of −1.5 to 1.5 mm/year for 2011–2014 (Fig. 3).

Although the displacement values are very low, close to the spatially consistent signal, we could identify a general uplift pattern located between the two river corridors that drain the city’s surface: Dâmboviţa and Colentina Rivers. This movement could be altered by a short-term trend of aquifers reloading and show a long-term trend possibly caused by a tectonic stress, as will be further discussed later.

To derive the long-term trends, we selected sufficiently large areas, where human intervention was likely to have imprinted short-term variations on the general displacement trend, using diachronic analysis of cartographic materials and historical evidence^32^,^33^. Distinguishing between short-term and long-term variations is essential for areas where ground displacement is very close to noise values. In order to differentiate natural evolution from human intervention, we selected areas located on all types of topographic features that can be found in Bucharest: alluvial plain, terraces, and interfluves. The working hypothesis was that differences in deformation patterns between the test zones and their surroundings will point out short-term trends superimposed on long-term trends. Areas that were significantly modified by humans through radical changes in land use, superimposed on the regional displacement trends, display reactions translated into higher instabilities that can be better detected by measuring instruments. Taking into consideration the working hypothesis that short-term variations could highlight the evolution trend of an area, we focused our analysis on ground displacements of industrial parks.

### Development Trends of Industrial Parks

**Figure 4:**
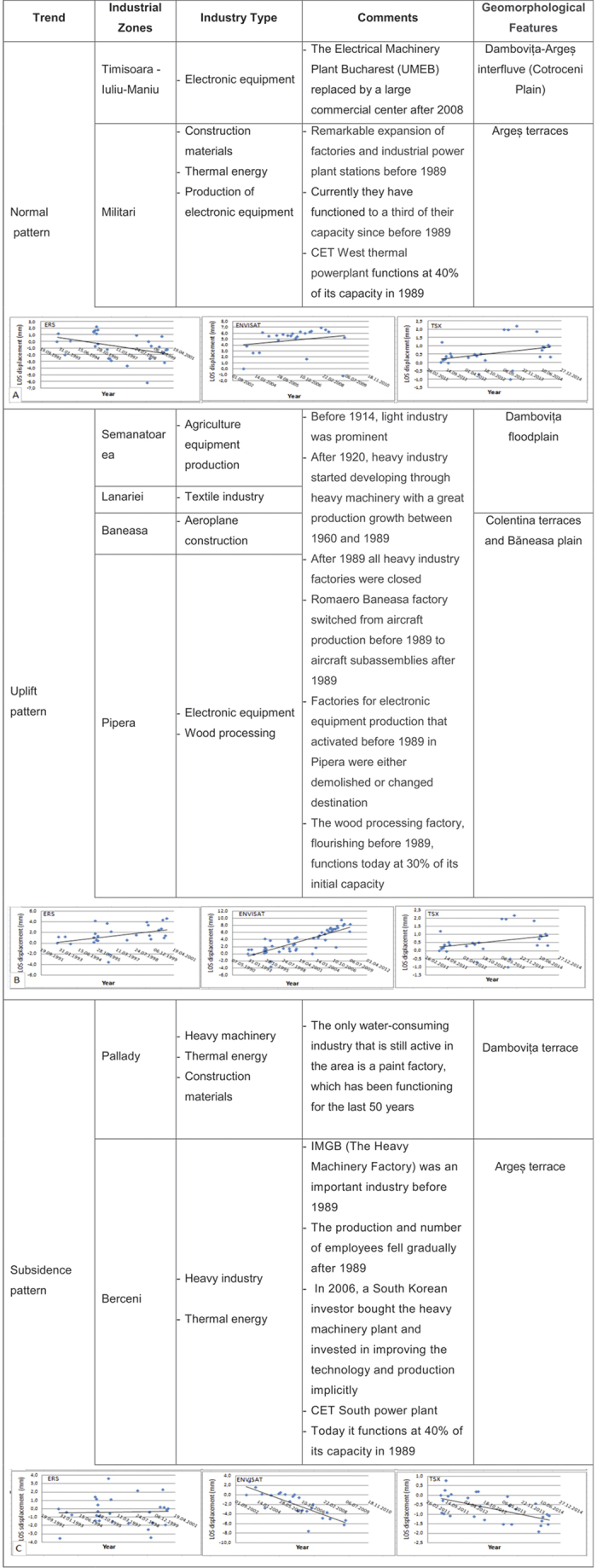
Dynamic trend and history of industrial parks.

At an international level, there are several studies that point to a direct relationship between groundwater extraction and subsidence in urban areas^12^,^34^,^35^,^36^. For Bucharest, we calculated the mean annual deformation rates in each time period for 20 large water-consuming industrial parks to identify the spatial evolution trends before and after 2000, when most of the industry was shut down (Fig. 4). Part of the results of ground displacement trends of industrial parks in Bucharest were presented in the *9*^*th*^ *International Workshop Fringe 2015*^21^.

The post-processing analysis of each PS time series was performed through statistical classification of the behaviours of each industrial area. The ground movements were interpreted on a geomorphological basis, according to the evolution of the groundwater levels and water use in time, and in relation to the history of the industrial parks^21^.

The outcomes emphasised three main patterns that characterise ground displacements in industrial parks over 1992–1999, 2003–2010, and 2011–2014: an ‘expected’ pattern, a continuous uplifting pattern, and a subsidence pattern.

We defined the ‘expected’ pattern as subsidence in 1992–1999 and uplift after 2003. Until 1989, the industrial parks were in operation, most of them relying on on-site groundwater extraction^37^, therefore causing a drop-in pore pressure that could result in a subsiding trend on the PS time series. After 2000, when more than half of the industry in Bucharest was shut down, these industrial parks showed a tendency to return to the zonal movement trend, developing compensatory higher positive displacement rates compared to their surroundings.

The continuous uplifting pattern is mainly characteristic of 10 industrial parks located in uplifting areas like the interfluves between Dâmboviţa and Colentina Rivers and on river accumulation forms in the Dâmboviţa River’s corridor.

The subsiding pattern (i.e., continuous subsidence) was noted for the Berceni area and for the Pallady industrial park. Both are located in the south-east of the city. These areas display a continuous subsidence trend, depicted in all three analysed satellite datasets. The subsidence in the two areas is tectonically imposed, as explained later in the paper, but the higher velocity values for the Berceni industrial park may also be caused by the fact that, during the time covered by our research, heavy machinery factories have continued being active, and investments have been recently made to increase production. In addition to the industrial activity, the Berceni industrial park is surrounded by residential areas that have dramatically expanded after 2000; a possible compaction of the ground could also have resulted from the pressure exerted by the new buildings’ weight.

### Displacement Trend Patterns

The evolution of landscape is, in many cases, nonlinear. In the last few years, it has also become possible to analyse and explain changes in topography using concepts associated with nonlinear dynamical systems^38^,^39^,^40^,^41^. Unfortunately, up to the present time, the study of nonlinear dynamic geomorphological systems has remained theoretical in geosciences. Observation techniques for ground displacements using multi-temporal InSAR analysis can reveal linear versus nonlinear evolution patterns in geomorphological features, especially in urban areas subject to a high density of coherent points in time.

In this paper, besides investigating the linear patterns identified in the PS InSAR analysis, we tested the presence/absence of nonlinear dynamics over selected industrial parks using SBAS InSAR. We also chose to use more than one method to test the topographic dynamic patterns to avoid spurious results related to errors and limitations of each procedure.

**Figure 5:**
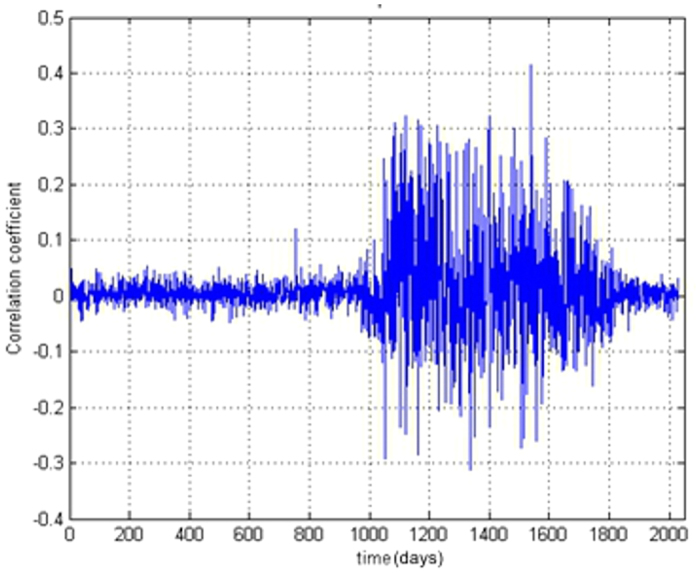
Correlation coefficients between displacement and temperature for the Berceni industrial park. Figure created in MATLAB^®^6.5.

Since ground displacements (and more generally geomorphological systems) are dynamic systems and their states can change with variation of external or internal drivers, we first analysed the correlation between displacements values from SBAS and weather conditions: daily temperature and precipitation over the period covered by the TSX data. Very low correlation coefficients were obtained with a mean value of approximately 0.2, with an isolated maximum positive correlation coefficient of 0.4 for the Berceni industrial park. Therefore, it appears that temperature and precipitation variations do not provide an explanation of ground displacement trends. Figure 5 is a graphical representation of the correlation coefficients series for the Berceni industrial park.

Secondly, we assumed that the spatial and temporal characteristics of the ground displacement data, like serial correlation, stationarity, noise level, linearity or nonlinearity, and long memory, may affect the stability and the performance of any classification. Each one of these measures and properties was analysed and the main achievements are presented next for each main pattern group found for the industrial parks on the basis of an example: (i) Militari for the ‘expected’ pattern, (ii) Lanariei for the continuous uplifting pattern, and (iii) Berceni for the subsiding trend. For more details of the related tools see Schreiber^42^.

To test nonlinearity, we applied a Brock, Dechert, and Scheinkman (BDS) test to our data for embedding dimensions of *m* = 2, 3, 4, 5 and 6. We used the quantiles from the small sample simulations reported by Brock *et al*.^43^ as approximations to the finite-sample critical values of our BDS statistics. The independent and identical distribution (i.i.d.) null hypothesis is rejected in all cases for yield changes. The BDS statistics clearly showed that there is no linear dependence in the data. The BDS test examines the null hypothesis of i.i.d. in the data against an unspecified departure from i.i.d. A rejection of the i.i.d. null hypothesis in the BDS test is consistent with some type of dependence in the data, which could result from a linear stochastic system, a nonlinear stochastic system, or a nonlinear deterministic system. That means that once the null is rejected, the displacement data will not be i.i.d. and some more complex studies will have to be implemented to obtain insights about the nature of the data.

Stationarity was also analysed, based on the unit roots test (e.g., Augmented Dickey-Fuller and Phillip-Peron, in Hamilton)^44^. Time series stationarity (i.e., invariance of statistical properties over time: constant mean, constant variance, and constant co-variance) is an important characteristic that may affect the classification of the time series and the fitted model stability and performance. For example, linear stationary time series can be easily classified and linear ARMA (Autoregressive Moving Average) models can be fitted to these data. For temporal observations where the variance is not constant over time or heteroskedastic, which are non-stationary, we can fit GARCH (Generalised Autoregressive Conditional Heteroskedastic) nonlinear models^44^. Moreover, nonlinear deterministic behaviour is also specific to stationary time series^42^,^45^.

We are mainly interested in stationary time series because many models and tools, deterministic and stochastic, have been developed for this kind of process^44^,^45^. The two main reasons why the statistician uses stationary time series are as follows: most statistical forecasting methods are based on the assumption that the time series is stationary, and in order to be able to obtain meaningful sample statistics, such as means, variances, and correlations with other variables, the series should be stationary.

Most of the analysed displacement time series were stationary or linear trend-stationary, which means that the time series varies along a linear trend. That is, the mean increases linearly with time, but the variance is constant in time. All trend-stationary time series were detrended, where by detrending we understand the process of removing the effects of accumulating datasets from a trend in order to obtain only the absolute changes in values and to allow potential cyclical patterns to be identified. The detrending is performed using regression and other statistical techniques. The new stationary time series were analysed in order to find the evolution pattern of the adjacent natural system.

**Figure 6:**
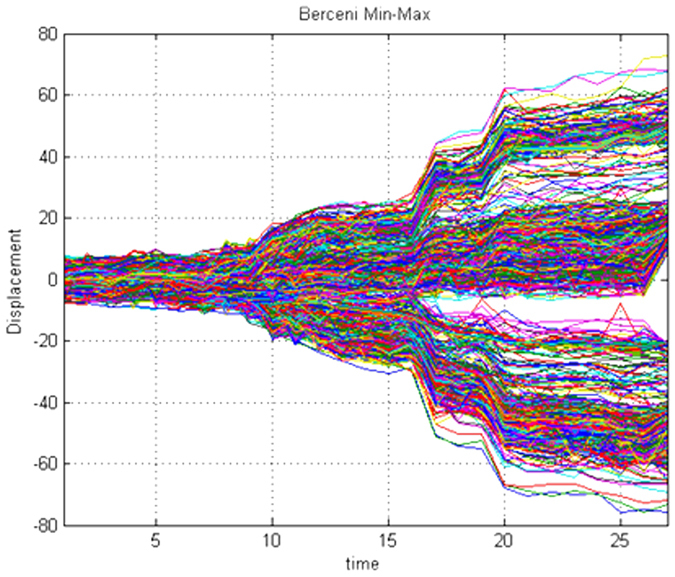
Min-max displacement time series for the Berceni industrial park, showing a selection of the 1000 most minimum and maximum time series from the dataset. Figure created in MATLAB^®^ 6.5.

A key challenge in Earth science research is the extraction of information from the huge spatio-temporal datasets generated by the data assimilation process (see for instance Min-Max time series for the Berceni industrial park in Fig. 6). These datasets comprise observations of extremely complicated multivariate processes. Thus, methods of analysis must be able to account for multiscale dynamical variability across different dynamical variables in space and time, account for various sources of error, and provide efficient dimension reduction. Principal component analysis (PCA) generates a new set of variables based on a linear combination of the original parameters. All the principal components are orthogonal to each other, so there is no redundant information. Considering that some variables are correlative, it is reasonable to select a few series with a higher confidence level as the state variables. In this study, we chose three or four state variables to establish the dynamical model of the evolution process of ground displacement.

The next step of the analysis was a two-dimensional data matrix. Each column represented the time series for a given location and each row represented a point in time. We performed a PCA on the data matrix and obtained the loadings and scores for the principal components. The loadings give the spatial pattern of the 1^st^ principal component; the scores give the variability of the 1^st^ principal component through time.

**Figure 7:**
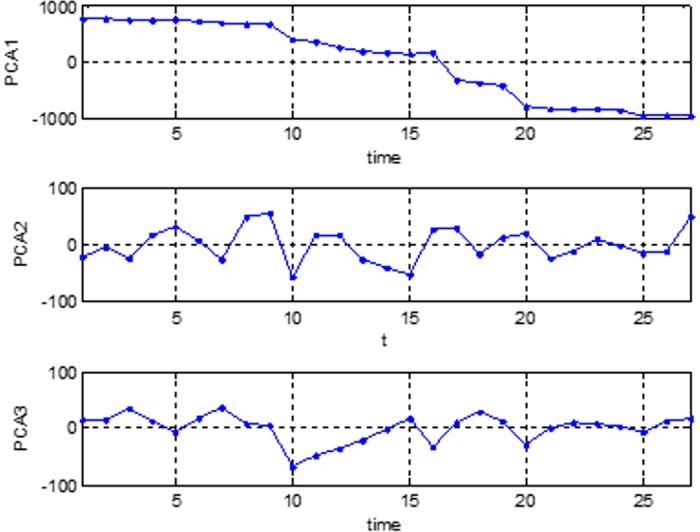
First three principal components for Berceni industrial park. Figure created in MATLAB^®^ 6.5.

Figure 7 shows the first three principal components for the Berceni industrial park. The obtained results for the three considered industrial parks are quite similar, as can be observed later in the final attractor representation.

**Figure 8:**
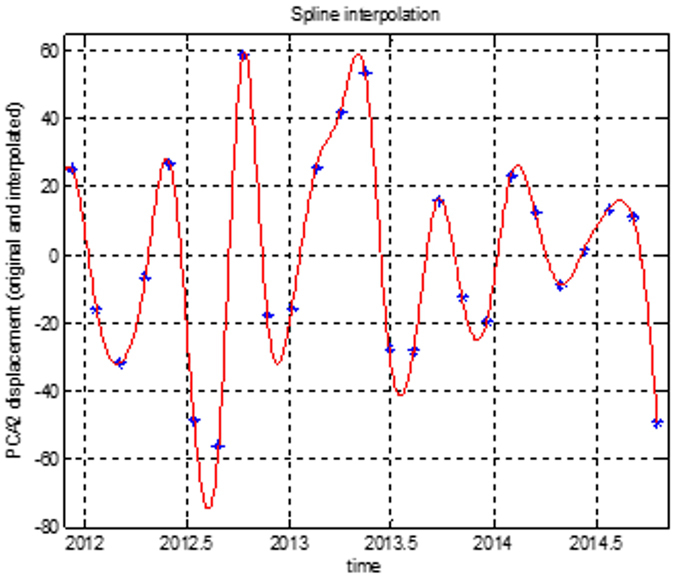
Spline interpolation for PCA time series, the Berceni industrial park. Figure created in MATLAB^®^ 6.5.

Natural processes on Earth often show a more complex and chaotic behaviour, therefore methods based on linear techniques may produce unsatisfactory results. New techniques for nonlinear data analysis derived from chaos theory have become quite popular over the last few years, being able to distinguish between regular and chaotic dynamics in a deterministic system. Unfortunately, in order to be able to apply nonlinear tools, a long-time series is needed. For this reason, a spline interpolation method was applied to all principal component series, for each one of the industrial park areas, in order to increase the number of observations. We decided to choose the spline interpolation after testing other approximation methods (linear, cubic, fast Fourier). All the nonlinear tools and mechanisms were applied to the displacement interpolated time series. Figure 8 shows an example of the spline interpolated time series for one of the PCA series for the Berceni industrial park. The stars are the original time series observation, and in grey (red) we have represented the spline-interpolated time series (5000 observations).

To describe nonlinear behaviour, different methods have been employed by defining, for example, scaling laws and fractal dimensions of natural features^40^,^42^,^46^. In this study, we applied the following nonlinearity/chaos theory techniques: the 0–1 chaos test, Takens Theorem to embed and reconstruct the phase space^47^, Hurst exponents and fractal dimension, Kolmogorov entropy, and Lyapunov exponents.

The usual test of whether a deterministic dynamical system is chaotic or non-chaotic involves the calculation of the maximum Lyapunov exponent λ. A positive maximum Lyapunov exponent indicates chaos, that is, if λ > 0, then nearby trajectories separate exponentially. A negative Lyapunov exponent corresponds to the existence of stable cycles and regions and if λ ≈ 0, then we have a random walk process. This approach has been widely used for dynamical systems whose equations are known. If the equations are not known or one wishes to examine experimental data, then λ may be estimated using the phase space reconstruction method of Takens by approximating the linearisation of the evolution operator. In contrast, the 0–1 chaos test does not depend on phase space reconstruction, but rather works directly with the time series given. In practice, the Gottwald-Melbourne 0–1 test for chaos output reduces to the following: the output result is near 0 for non-chaotic data and near 1 for chaotic data^48^.

The Hurst exponent, proposed by H. E. Hurst^49^ for use in the fractal analysis, has been applied to many research fields, and recently in Earth science also^41^,^46^,^50^. The Hurst exponent provides a measure of the long-term memory and the fractality of a time series and, consequently, it can be used as a numerical estimate of the predictability of a time series. It is defined as the relative tendency of a time series to either regress to a longer term mean value or to cluster in a direction. The values of the Hurst exponent range between *0* and *1*. Based on the Hurst exponent value *H*, a time series can be classified into three categories: *H* = *0*.*5* indicates a memory-less time series, with neither short-term nor long-term correlation between states, typical of uncorrelated stochastic processes as white noise; *0* < *H* < *0*.*5* indicates anti-persistence: increasing trends will be followed by decreasing ones, or vice versa, and this behaviour tends to be dominant for H → 0; and *0*.*5* < *H* < *1* indicates persistence. In this case, there is only one persistent trend typical of processes where diffusion is faster than simple Brownian motion.

In this paper, phase space reconstruction, the 0–1 chaos test and Lyapunov exponent methods were employed for detecting chaos, whereas the Hurst exponent was performed to identify scaling characteristics. These methods have a dual role, in the sense that we can use them to distinguish between stable cycles and chaotic behaviour in a low-dimensional nonlinear deterministic systems, but also, and more importantly, to distinguish a chaotic deterministic process from a (linear or nonlinear) stochastic one^42^,^45^.

For all the 0–1 chaos tests we ran and for all types of data considered, we always rejected the null of a chaotic process in favour of a nonlinear cyclical or quasi-cyclical dynamic. The estimation of the maximum Lyapunov exponents, based on Wolf *et al*.’s methodology^51^, was more controversial, since in some cases we obtained positive values in the interval [1,1.5] and in other situations negative values close to zero. The last case is more concordant with the 0–1 chaos test.

**Figure 9:**
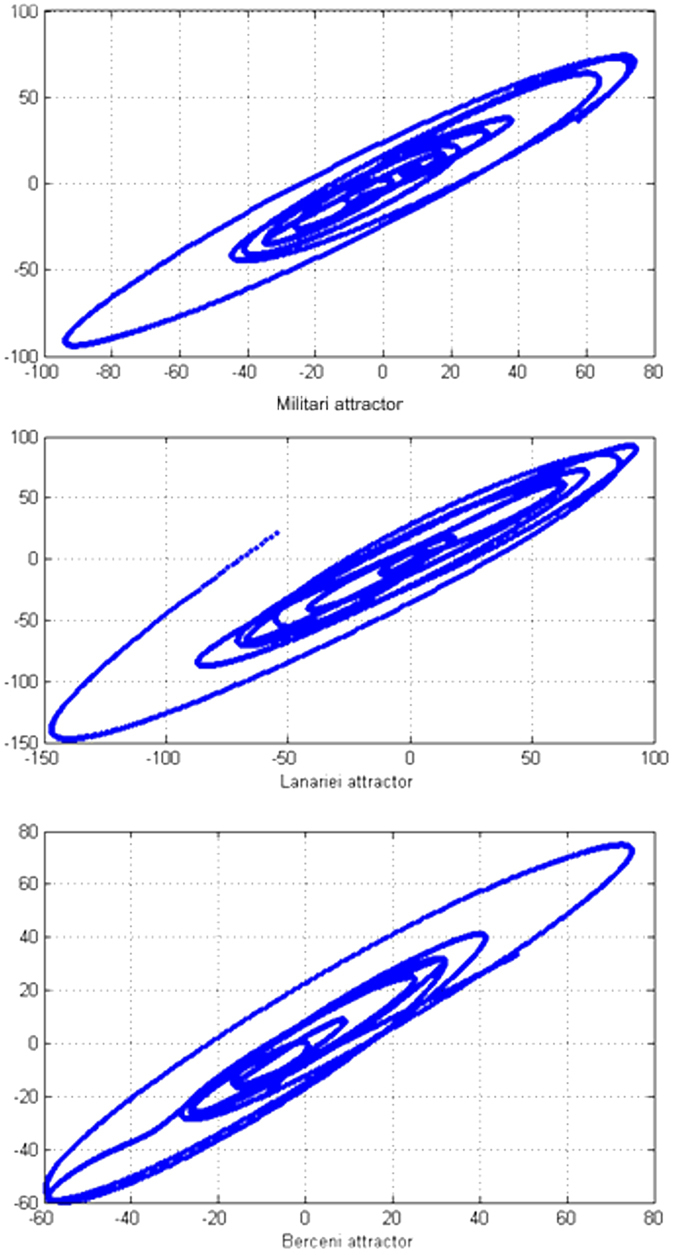
Attractors for the three considered industrial parks. Figure created in MATLAB^®^ 6.5.

The phase space reconstruction based on Takens’ theorem suggested for all situations deterministic nonlinear attractors with very well defined patterns. Embedding dimensions and optimal delays were determined by the usual procedures. Figure 9 illustrates the attractors for each one of the three considered representative industrial parks. An attractor represents a set of points in the state space that are invariant under the system dynamics, which means that asymptotically all the trajectories will settle down on the attractor (fitting the final shape of the dynamics). If the attractor dimension is fractal (i.e. a non-integer number), then we have a strange attractor, as in the analysed situations.

All displacement time series show a very persistent long memory behaviour characterised by Hurst exponents with values in the interval [0.9,1]. This suggests a fractal Brownian motion or a periodic (quasi-periodic) nonlinear deterministic process. Taking into account the results, we are inclined to opt for a nonlinear periodic process as being the main model for ground displacement dynamics in Bucharest, based on the study of large industrial parks and historical clay pits spread in the meadows and on the interfluves between the Dâmboviţa and Colentina Rivers.

## Discussion: A tectonic hypothesis

The InSAR TSX data show uplift movements in an area that seems to extend on a northwest - southeast oriented domain that is roughly located between the Dâmboviţa and Colentina Rivers’ corridors and coincides with the location of most of the buildings classified to pose seismic risk because of compromised structures (i.e. fissures/fractures). Unfortunately, the Quaternary unconsolidated sand, gravel, and plastic clayey rocks do not retain any visible markers (faults, fractures, or fissures) that could offer further insight into this matter. In order to investigate these displacements further, we continued with the working hypothesis that ground movements should leave traces in the manmade structures above, especially in the taller buildings with deeper foundations. The House of People (‘Casa Poporului’), the Palace of the Romanian Parliament is by far the largest and the heaviest building in Bucharest (and in Europe), whose structure is reputed to measure not only 86 m above ground level, but also 92 m in depth. Considering the depth of its foundations and the large surface contact with the geological substrate (we estimate a total vertical surface contact of ~93840 m^2^ and a horizontal surface contact of ~64800 m^2^ at a depth of 92 m below surface), the stress generated by ground displacements is expected to be transferred to the structure of the building. The fact that the Palace of the Parliament lies in the uplift domain makes it an ideal candidate for our investigations.

**Figure 10:**
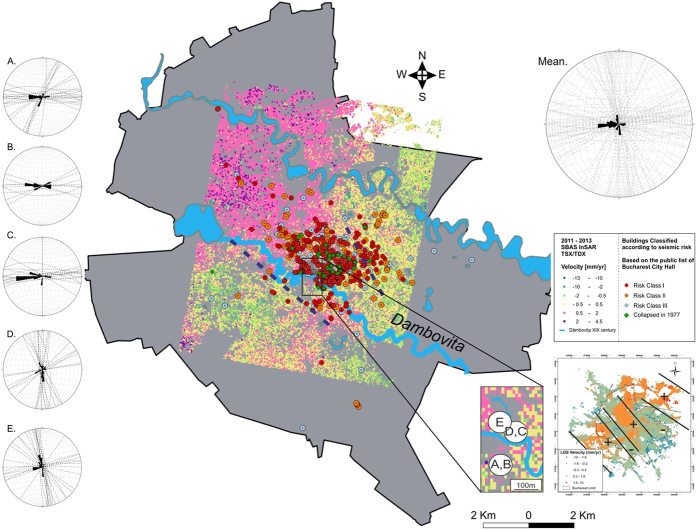
PSInSAR applied to TSX satellite data and rose diagrams showing the orientation of fissures and fractures measured in buildings. (**A**) Palace of the Romanian Parliament, (**B**) surroundings, (**C**) Town Hall, (**D**) National Archives, (**E**) Lazăr College and surroundings. The box in the lower right shows the location of the measurement points. Red and orange circles represent individual buildings classified to pose seismic risk, while green circles are the buildings that collapsed during the 1977 earthquake. The blue dashed lines suggest a possible NW-SE oriented domain. On the right map, at least three uplift domains can be inferred, all of them part of a NW-SE transpressional system. Figure created in Esri^®^ArcMap™ 10.2.

The building was searched for fissures and fractures that can be followed in at least two orthogonal planes of the same volume, or in the same plane but in two different volumes (a volume being a wall, a column, and staircase). The azimuth of the discontinuities (fissures and fractures) was measured with a geological compass. By using the same methodology, we have enlarged the study area towards the north, choosing 3 additional historical buildings and their surroundings: the Town Hall, National Archives, and Lazăr College. The results are plotted in rose diagrams, in Fig. 10.

The Palace of the Parliament (Fig. 10A) shows a dominant east-west oriented set of fissures (1) that can also be traced in the surroundings (Fig. 10B) and in the Town Hall (Fig. 10C). In the National Archives (Fig. 10D) and Lazăr College (Fig. 10E) this set of fissures is replaced by a southwest-northeast, respectively SWW-NEE fissure system (2). The Town Hall, National Archives, and Lazăr College also show an approximately north-south-oriented set of fissures (3) that are not observed in the Palace of the Parliament nor its surroundings. Here, a northeast-southwest system is observed (4). The mean plot indicates that the dominant fissure systems are east-west (1) and ~north-south (NNW-SSE) (3).

Systems (1) and (3) are actually quasi-perpendicular on the buildings’ facets. This is not unexpected, since they seem to exploit the directions of lateral least structural resistance (the vertical contact plane between bricks). This could be indicative of a northwest-southeast oriented stress field that is transmitted in the buildings, where it dissociates in north-south and east-west stress fields. Interestingly, the locations of the buildings classified as prone to seismic risk and the locations of those that collapsed during the 1977 earthquake also suggest a northwest-southeast ‘risk domain’ (Fig. 10, map). Set (2) could also be a result of the same stress field. Set (4) is completely different to the others and was observed only in the Palace of the Parliament. This is difficult to interpret and could simply be a result of a structural flaw.

Our working hypothesis at this moment is that the uplift recorded by the TSX data is the result of tectonic stress fields generated by transpressional movements related to the Intra-Moesian Fault system, located less than 40 km east of Bucharest^52^. The Intra-Moesian fault system has a northwest-southeast trend, which coincides with our presumed transpressional domain. This suggests that Bucharest has been built on peripheral components of this fault system that are currently active.

## Conclusions

The aim of our study was to capture long-term patterns of ground displacements using single polarised SAR historical and recent satellite data, PS InSAR, and SBAS InSAR techniques for a very crowded and dynamic city of Europe: Bucharest, the capital city of Romania. The selection of test zones was made considering the human impact criteria: large industrial parks spread in the meadows and on the interfluves between the Dâmboviţa and Colentina Rivers. Large industrial parks constructed during the Communist Era on various landforms that can be found in Bucharest represent ideal test sites for identifying long-term evolution patterns through comparative analysis with the evolution trends of their neighbouring areas. The industrial parks display long-term evolution patterns that have been perturbed by human interaction in the past, but are now recovering towards their initial behaviour. Natural long-term trends can be highlighted by contrast with overlaying short-term patterns caused by human interaction.

A wealth of statistical and numerical tests was employed to find evidence of nonlinear dynamics and asymptotic attractors. As a first step, a data reduction method was necessary and we opted for PCA, reducing the huge samples to 3 or 4 time series containing all decisive information of the corresponding sample. Stationarity (by unit root tests) and linearity (by BDS test) was analysed. Once we eliminated the linearity assumption and detrended the nonstationary data, we applied a spline interpolation in order to increase the number of observations in the time series. For these new time series, we considered several tools for searching for nonlinear chaotic or periodic dynamics.

Phase space reconstruction, the 0–1 chaos test and Lyapunov exponent methods were employed for detecting chaos, whereas the Hurst exponent was performed to identify scaling characteristics. These methods have a dual role, in the sense that we can use them to distinguish between stable cycles and chaotic behaviour in a low-dimensional nonlinear deterministic system, but also, and more importantly, to distinguish a chaotic deterministic process from a (linear or nonlinear) stochastic one.

The phase space reconstruction based on Takens theorem suggested for all situations, deterministic nonlinear attractors with very well defined patterns. All displacement time series show a very persistent long memory behaviour characterised by Hurst exponents with values in the interval [0.9,1]. This suggests a fractal Brownian motion or a periodic (quasi-periodic) nonlinear deterministic process. Taking into account the results of other implemented tests, we are inclined to opt for a nonlinear periodic process that is the main model for ground displacement dynamics in Bucharest, which we assume to have as possible asymptotic attractors an active northwest-southeast oriented transpressional system. If future studies support the idea that Bucharest is partially built on an active transpressional system, significant geodynamic and civil engineering implications may result. This may also be the reason for the distribution of the buildings classified as posing a seismic risk.

## Additional Information

**How to cite this article:** Armaş, I. *et al*. Long-term ground deformation patterns of Bucharest using multi-temporal InSar and multivariate dynamic analyses: a possible transpressional system? *Sci. Rep.* **7**, 43762; doi: 10.1038/srep43762 (2017).

**Publisher's note:** Springer Nature remains neutral with regard to jurisdictional claims in published maps and institutional affiliations.

## Figures and Tables

**Figure 1:**
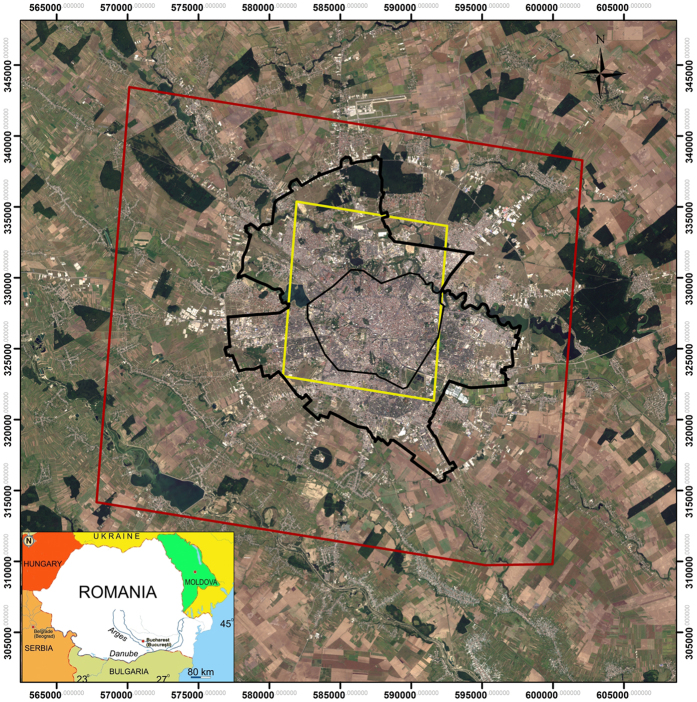
The red polygon indicates the extent of the study area. The yellow polygon represents the extent of the focused TerraSAR-X PS InSAR analysis on industrial parks. The administrative limits of the historical and the present-day city are represented in grey and black lines, respectively. City limits were drawn after historical and recent maps. The extents of the areas that were processed using PS and SBAS methods were generated using SARMAP’s SARscape^®^ software package 5.1. Map created in Esri^®^ArcMap™ 10.2. Base map: Landsat-8 scene from 26/08/2015, path 183, row 183, downloaded for free from Earth Resources Observation and Science (EROS) Center, USGS (http://earthexplorer.usgs.gov/; accessed 02/01/2016).
